# Pathology

**Published:** 1890-10-25

**Authors:** 


### PATHOLOGY.

Dr. Savill, in his pathological demonstration, described at considerable length two cases, both of great interest, not only pathologically, but also clinically. The first was that of an old man, J. H., aged 76. Clinically he was of interest, in that in his life-time he had had various diseases which tend to shorten life, and pathologically, he hardly had a normal organ in his body. Life itself was stated to be the equilibrium between the external and the internal conditions of the body. Dr. Savill felt disposed to define life not only as the equilibrium between the external and the internal conditions, but also as the equilibrium between the several organs of the body itself. The patient above-mentioned was admitted into the infirmary in January last. His father died at 78. There was nothing special to be made out of his family history. He had an attack of gout forty years ago—that is, at thirty-six; syphilis at seventeen. He then attended the Lock Hospital, and now had a typical syphilitic tongue, as shown by the heaping up of the epidermis. He used to drink freely both beer and spirits, especially the latter. He continued his occupation as a boot-maker till July, 1889, when he became paralyzed on the left side, the arm being first affected, and then a few days after the leg. There was never loss of consciousness. On admission he had marked left hemiplegia, with slight stiffness, but no loss of sensation. The erector spinæ was less prominent on the right than on the left side; no albumen in the urine. The arteries were thick and tortuous, the pulse slow and slightly irregular. He had a bronchial attack shortly before death, and died with symptoms of pulmonary hypostases. Post-mortem—The lungs were emphysematous at their edges, and there was evidence of old bronchitis with some fibrosis, together with patches of broncho-pneumonia. The heart showed brown atrophy of its muscles, some atheroma of the aorta, and the coronary arteries were calcareous. Most of the arteries showed typical specimens of atheromatous changes. The general pathology of atheroma was then described. How it affected the larger vessels was patchy and asymmetrical, the changes started beneath the intima, and from here they extended into the muscular layer; the process was essentially a low form of inflammatory deposit. The consequences of these changes in the vessels were as follows: (1) A diminution in nutrition in the parts supplied by the vessel; (2) total obliteration of the affected vessel; (3) weakening of the walls, leading to aneurysm; (4) emboli from separation of the atheromatous deposits. In other words, the changes, as a whole, consisted in the transformation of an elastic tube into a rigid rod, and it was the rigidity of the vessels that gave rise to the accentuated second sound of the heart so common in old people. Atheroma was then compared with syphilitic diseases of the vessels. The latter was first discussed by Dr. Wilks, and later a German, named Hubner, described it at great length, and described a granuloma in the walls of the vessels. Syphilis, when it affects the vessels, differed from atheroma in the following ways: (1) Its preference for the smaller vessels; (2) nearly always goes all round the lumen of the vessel; (3) the new tissue formed does not degenerate, but becomes vascular, that is organises; (4) a tendency to progressive increase.

Dr. Savill returned now to the case he was describing. The liver was slightly fatty and cirrhotic, the spleen was normal, the kidneys were practically normal, weighing six and six and a-half ounces respectively, with some slight cloudiness of the secreting epithelium of the tubules. A French classical writer has said that a man is as old as his arteries, but in this case a man appeared to be as old as his kidneys. Dr. Savill believed that it was not so much a diminution in the blood supply as the poisoned blood which shortened life. The brain showed a lesion in the right hemisphere between the corpus striatum and optic thalamus, revealing some softening in this area. In the cord nothing was demonstrable, but one would have expected to find, commencing degeneration down the anterior pyramidal tract of the same side, and of the lateral tract of the opposite side.

The second case was that of a man, H.A., aged 50, who was admitted on September 24th, 1890, and who died on October 16th. The history he gave was: He had had syphilis, and some accident in early life. His present illness began gradually in the spring of this year with loss of appetite, weakness, then pain in the legs, then weakness and feeling of numbness in the legs, that is to say, the symptoms of "progressive paraplegia." The pain increased, and he was admitted into St. Mary's Hospital for a time. Gradually he became completely paralysed with bedsores over the sacrum, and then marked atrophy of the muscles of the legs, and died. Post mortem,—the liver was enlarged, and weighed 83 ounces and contained deposits of new growths. These were brain-like in consistence and highly vascular. The diaphragm also contained deposits, but these were upon its upper surface, likewise the pleuræ and external surface of the pericardium. Kidneys: the one contained a large mass of growth, and the other was filled with a disseminated deposit throughout. Lungs: the upper lobe of the right was pneumonic, passing into grey hepatisation; weight 36 ounces. At this point the lecturer remarked upon the frequency of patients with nerve diseases dying from pulmonary troubles. The aorta had some patches, probably of atheroma. The brain was normal. Spinal cord: In the region of the lower dorsal region there was a nodular deposit external to the theca, and the cord itself revealed about three inches of softening due to the pressure of the growth. There was also a growth in front of the spine on the right sides of the bodies of the last three vertebræ. The question as to the original seat of the growth was a very hard one, the growths in the liver were larger and firmer than the others, but considering how rarely, if, indeed, primary growths in this organ ever occurred, it was difficult to say that this was the organ first affected; and, unfortunately, in this case the stomach had not been examined. The nature of the growths, as revealed by the microscope, showed them to be medullary or encephaloid carcinoma.

